# Prevalence of Elevated CK Levels, Myositis-Specific and Myositis-Associated Antibodies, Myositis, and Other Neuromuscular Diseases in Myasthenia Gravis Patients—Experience from an Eastern European Tertiary Center

**DOI:** 10.3390/jcm14072449

**Published:** 2025-04-03

**Authors:** Márk Kozák, Edina Kovács, Melinda Nagy-Vince, Attila Tóth, Judit Boczán

**Affiliations:** 1 Department of Neurology, Faculty of Medicine, University of Debrecen, 22 Móricz Zsigmond krt., 4032 Debrecen, Hungary; kozak.mark@med.unideb.hu; 2 Department of Developmental Neurology, Szent Margit Budapest Hospital, 132 Bécsi út, 1032 Budapest, Hungary; pappne.dr.kovacs.edina@sztmargit.hu; 3 Division of Clinical Immunology, Faculty of Medicine, University of Debrecen, 22 Móricz Zsigmond krt., 4032 Debrecen, Hungary; nagy-vincze.melinda@med.unideb.hu; 4 Division of Clinical Physiology, Department of Cardiology, Faculty of Medicine, University of Debrecen, 22 Móricz Zsigmond krt., 4032 Debrecen, Hungary; atitoth@med.unideb.hu

**Keywords:** myasthenia gravis, myositis, idiopathic inflammatory myopathy (IIM), elevated CK levels, MSA, MAA, vitamin D

## Abstract

**Background:** Myasthenia gravis (MG) and idiopathic inflammatory myopathy (IIM) are autoimmune diseases that affect the musculoskeletal system. The association of the two diseases is rare. Their management is different, so it is important to recognize the concomitant presentation. **Methods:** In this cross-sectional study, we study the presence of CK elevation, myositis-specific and myositis-associated antibodies (MSA/MAA), and vitamin D levels in a cohort of 101 MG patients. Electromyography, limb magnetic resonance imaging (MRI), and, in some cases, muscle biopsy were performed when IIM was suspected. We reviewed the patients’ medical records to access the results of these tests if they had been performed previously. **Results:** CK elevation was detected in 10 patients (9.9%). We identified one case of anti-Jo-1 antibody-positive polymyositis and two cases of possible myositis. MSA/MAA antibodies were not found in the patients with high CK levels, except for the one with anti-Jo-1-positive IIM. One patient with elevated CK levels had an overlapping muscular dystrophy. MSA/MAA antibodies were detected in 19 patients (18.8%). A total of 37% had high-titer antibodies and concomitant systemic autoimmune diseases, while 63% had low-titer antibodies, most of whom had no systemic autoimmune disease. Low serum vitamin D levels were found in 67.3% of patients. Comparison of myasthenia gravis composite (MGC) scores between patients with low and normal vitamin D levels did not show a statistically significant difference. **Conclusions:** Our results may raise awareness among neuromuscular specialists caring for MG patients of the possibility of associated myositis or other neuromuscular diseases and the need to assess vitamin D levels. Although deficiency was frequent, its impact on MG severity remains unclear, necessitating further investigation into its immunological relevance.

## 1. Introduction

Myasthenia gravis (MG) and idiopathic inflammatory myopathy (IIM) are rare autoimmune diseases affecting the skeletal muscle. Both conditions result in muscle weakness, making differentiation difficult when they occur simultaneously [1].

In MG, autoantibodies target components of the neuromuscular junction, most commonly the acetylcholine receptor (AChR, 85% of patients) [1]. In anti-AChR antibody-negative cases, antibodies against muscle-specific kinase (MUSK, 6% of patients), lipoprotein receptor-related peptide 4 (anti-LRP4), ryanodine receptor, and titin have been found [2,3]. Pathological lesions of the thymus play an important role in the pathogenesis of MG, being present in more than 50% of cases [3,4].

Idiopathic inflammatory myositis includes four main clinical groups: dermatomyositis (DM), polymyositis (PM), immune-mediated necrotizing myopathy (IMNM), and sporadic inclusion body myositis (IBM). In IIM, myositis-specific (MSA) and myositis-associated (MAA) antibodies can be beneficial in the classification of myositis [5].

MG usually presents with fatigability and fluctuating muscle weakness affecting the ocular, bulbar, axial, and limb muscles. On the other hand, IIM usually manifests as a non-fluctuating, progressive generalized and bulbar muscle weakness, often associated with myalgias [6]. From a clinical point of view, the most important difference between MG and IIM is the ocular symptoms, which are frequent in MG but practically absent in myositis [3,4]. IIM can be associated with systemic autoimmune diseases, e.g., arthritis, interstitial lung disease, etc. Decremental response at repetitive nerve stimulation (RNS) and increased jitter and blocks at single-fiber electromyography (SF-EMG) are characteristic of myasthenia. By contrast, fibrillation potentials with myogenic pattern on electromyography (EMG) are typically seen in IIM. Inflammation of the skeletal muscle can also be confirmed in short tau inversion recovery (STIR) sequences on magnetic resonance imaging (MRI). In addition to testing for autoantibodies, serum creatine kinase (CK) testing is recommended in each case with muscle weakness. CK is usually elevated in myositis, whereas it is typically in the normal range in MG. In the case of suspected myositis, a muscle biopsy provides a definitive diagnosis, showing infiltrating lymphocytes in the tissue. In MG biopsies, it is usually negative, though lymphorrhagias may occur [7,8].

Low serum vitamin D levels (<75 nmol/L are thought to play a significant role in the activity of many chronic autoimmune diseases, such as systemic lupus erythematosus (SLE), rheumatoid arthritis (RA), and multiple sclerosis (MS) [9]. Patients with IIM have lower serum vitamin D levels, too [10]. Several publications have reported lower serum vitamin D levels in patients with myasthenia [9,11,12,13,14]. Vitamin D deficiency is associated with impaired immune tolerance, increased inflammation, and reduced muscle regeneration, contributing to autoimmune diseases, including inflammatory myopathies [9]. Vitamin D receptors in muscle cells further support its role in maintaining muscle integrity and modulating immune responses [9]. An association between vitamin D deficiency and elevated CK levels, as well as the presence of MSA/MAA antibodies in inflammatory myopathies, has been suggested by prior research [5].

It is important to recognize the coexistence of MG and IIM. Recognizing such cases is crucial, as the treatment approach for myasthenia gravis differs from that of myositis. In MG, the first-line therapy is pyridostigmine, an ACh-esterase inhibitor that enhances neuromuscular transmission at the NMJ. In contrast, the primary treatment for myositis is corticosteroids. While both conditions may require immunosuppressive therapy in case of a flare-up of the disease, sudden administration of high-dose corticosteroids in MG— unlike in myositis—can lead to the severe worsening of muscle weakness, potentially triggering a myasthenic crisis [1]. In addition, a steroid dip has also been reported at doses typically used for MG. Recently, numerous cases with coexisting MG and IIM have been published after immune-checkpoint inhibitor (ICI) treatment of patients with malignancies [15]. On the other hand, the coexistence of the two diseases in ICI naïve patients seems to be rare—approximately 70 cases have been published in the literature (mainly case reports and a few case series) [16]. We have limited data about the prevalence of IIM among MG patients [1,3,4,6]. Though a few case studies report elevated CK levels in MG patients due to IIM or other neuromuscular diseases, data about the prevalence of elevated CK levels alone (either due to IIM or any other etiology) in a population of MG patients are scarce [17,18,19].

In this cross-sectional study, we aim to investigate the prevalence of elevated CK levels, MSA and MAA antibodies, and vitamin D deficiency in MG patients treated at our neuromuscular center. Additionally, we explore the potential relationship between these biomarkers to determine whether some MG patients exhibit features of overlapping neuromuscular disorders, particularly inflammatory myopathies. This research may contribute to a better understanding of disease heterogeneity in MG and provide insights into novel diagnostic and therapeutic approaches.

## 2. Materials and Methods

### 2.1. Patients

A total of 101 patients with a confirmed diagnosis of MG who visited the regional tertiary Neuromuscular Center in the Department of Neurology, University of Debrecen, Hungary, between 3 December 2019, and 17 March 2022, were included in this study. All participants were 18 years of age or older and the diagnosis of MG was previously based on clinical, immunological, neurophysiological, and pharmacological criteria according to international recommendations [20]. The study followed a non-randomized, cross-sectional design with both retrospective and prospective components. Consecutive MG patients visiting the center during the study period were sequentially enrolled, ensuring a representative sample of our MG patient population. All study procedures were approved by the Regional and Institutional Ethics Committee, Clinical Center, University of Debrecen (DE RKEB/IKEB 5313-2019). The study conforms to The Code of Ethics of the World Medical Association (Declaration of Helsinki). All examinations were undertaken with each subject’s understanding and written consent.

### 2.2. Neurophysiological Studies

Needle EMG was performed either at baseline or during the study period when myositis was suspected. At least three muscles were examined (deltoid, lateral vastus, and anterior tibial muscles). During their former diagnostic workup for MG, most of the patients underwent at least one neurophysiological study (RNS, SF-EMG).

### 2.3. Neuroimaging Study

Proximal limb muscles were examined using a 3T MRI scanner following the recommended protocol [21]. T1-TSE and STIR sequences were analyzed. Fibro-fatty replacement, muscle atrophy, and edema/inflammation on STIR sequences were determined.

### 2.4. Muscle Biopsy

Muscle biopsies were performed in selected cases where myositis or other neuromuscular disorders were suspected. The biopsy sites (deltoid or vastus lateralis muscle) were chosen based on clinical presentation and MRI findings. Tissue specimens were processed using standard histopathological techniques.

### 2.5. Serologic Studies

The patient’s sera were tested for CK and vitamin D levels at the university’s central laboratory (lower cut-off level of vitamin D was considered 75 nmol/L). Serum CK levels were measured using an automated enzymatic assay, with a reference range of 40–200 U/L. The presence of MSA and MAA antibodies was determined using a line blot test kit (Euroimmun, Lubeck, Germany) at the Department of Clinical Neurophysiology, University of Debrecen, according to the manufacturer’s instructions. The test included 16 different antigens: Mi-2α, Mi-2β, TIF1gamma, MDA5, NXP2, SAE1, Ku, PM-Scl100, PM-Scl75, Jo-1, SRP, PL-7, PL-12, EJ, OJ, and Ro-52. The evaluation of antibodies was based on the color intensity of the test, as suggested by the manufacturer (negative (−), borderline (+/−), single positive (+), double positive (++), or triple positive (+++)). In our analysis, single positive, double positive, or triple positive were considered positive, while negative and borderline cases were considered negative. The performance and evaluation of the line blots were uniform and impartial throughout the tested period.

### 2.6. Myasthenia Gravis Scales

Upon entering the study, the severity of myasthenia gravis was assessed using the *Myasthenia Gravis Foundation of America (MGFA) Clinical Classification*, which categorizes patients into five main classes (I–V) based on the severity and distribution of muscle weakness [18]. Additionally, disease burden was quantified using the myasthenia gravis composite (MGC) score, a validated tool involving both physician-based and patient-reported measures to assess the severity across multiple domains, including ocular, bulbar, respiratory, and limb involvement [19].

### 2.7. Classification of Patients for IIM

Patients with suspected myositis were evaluated by the online calculator for EULAR/ACR classification criteria for adult and juvenile idiopathic inflammatory myopathies (http://www.imm.ki.se/biostatistics/calculators/iim, accessed on 2 April 2025) maintained by the Unit of Biostatistics, Karolinska Institute, Stockholm, Sweden) [22].

### 2.8. Statistical Analysis

The MGC scores of patients with normal and elevated serum CK, positive and negative MSA/MAA antibodies, and low and normal vitamin D levels were tested with the Kolmogorov–Smirnov test. Since none of the groups in the pairwise comparison showed a normal distribution for either set of data (alpha was not <0.05 for either group), we used the Mann–Whitney test for comparison of MGC scores in these groups.

## 3. Results

A total of 101 patients treated between 3 December 2019, and 17 March 2022, at the Neuromuscular Outpatient Center were included in the study. The cohort consisted of 27 males (26.7%) and 74 females (73.2%). The median age of the patients was 57 years [IQR: 45–70], with a median MG onset age of 43 years [IQR:30–57] and a median disease duration of 7 years [IQR: 2.0–17]. Regarding MG subtypes, 68 patients (67.33%) had generalized MG, 18 patients (17.82%) had bulbar MG, and 15 patients (14.85%) had ocular MG. A total of 67 patients harbored AChR antibodies, and four were positive for MUSK antibodies. Fourteen patients were seronegative. In 16 patients, the diagnosis was based on electrophysiological tests conducted in the past; in these cases, antibody tests were not performed. Six patients had thymoma, and 39 patients had thymic hyperplasia (Table 1).

### 3.1. Elevated CK Levels

Of the 101 patients studied, 10 (9.9%) had elevated serum CK levels at the time of study entry (Table 2). Seven patients were AChR antibody positive, two were seronegative, and none were MUSK positive. AChR or MUSK antibody test was not performed in one patient (the RNS test was positive in her case). In six patients, CK levels did not exceed twice the upper limit of normal (ULN) and were normalized in three of them upon follow-up (patients No. 3, 6, and 7). In two patients (patients No. 8 and 10), CK was mildly elevated, and in patient No. 9 it exceeded 2×ULN upon follow-up. EMG showed mild myopathic changes in patient No. 3, while it gave normal results for the other patients. Since the MRI gave a normal result in patient No. 9., and the online calculator for IIM (http://www.imm.ki.se/biostatistics/calculators/iim, accessed on 2 April 2025) did not suggest myositis, a biopsy was not performed in this case. MRI and muscle biopsies were not performed in the other cases, either. None of these patients harbored MSA or MAA antibodies.

Repeated serum CK elevation exceeding twice the ULN was observed in four additional patients (patients No. 1, 2, 4, and 5). Electromyography showed signs of myositis in patients No. 1 and 5. A myopathic pattern was observed in patients No. 2 and 4. MRI examination suggested myositis in patients No. 1 and 4, fibro-fatty replacement in patient No. 2, and was normal in patient No. 5. Muscle biopsy confirmed myositis in patient No. 1, showed dystrophic signs in patient No. 2, myopathy in patient No. 4, and mild myasthenia-related abnormalities in patient No. 5. Serologic tests confirmed the presence of Jo-1, PM/Scl-75, and Ro52 antibodies in patient No. 1. The rest of the patients in this group did not harbor MSA/MAA antibodies. Patient No. 4 harbored anti-GAD65 antibodies. The online calculator for IIM suggested definitive IIM in patient No. 1 and probable myositis in patients No. 4 and 5. Comparison of the MGC scores of patients with normal and elevated serum CK levels did not show a significant difference between these groups (median [IQR]: 4.0 [1.0–7.0] vs. 3.5 [2.25–7.0], respectively, *p* = 0.9841).

#### 3.1.1. Patient No. 1

A 32-year-old female presented with a history of polyarthritis, Raynaud phenomenon, myalgia, and elevated CK levels (maximum 10,000 U/L) since 2017. An EMG, a limb MRI, and a vastus lateralis muscle biopsy confirmed myositis. Serological testing revealed positive anti-Jo-1, anti-PM/Scl-75, and anti-Ro52 antibodies. A diagnosis of anti-synthetase syndrome was established. She was treated with high-dose steroids and methotrexate, an approach that improved her symptoms. HRCT showed a persistent thymus, but no alveolitis or fibrosis. AChR antibodies were detected in her serum (0.56 nmol/L, cut-off: 0.4 nmol/L). The neurological examination conducted in 2017 was unremarkable, and RNS testing was negative. At that time, she had been receiving steroids and methotrexate for 4.5 months. Due to myositis exacerbation in 2018, methotrexate was switched to cyclosporine. In 2019, she developed fatigable paresis in the neck flexors and proximal limb muscle weakness, which improved with pyridostigmine (60 mg twice daily). Thymectomy was recommended. Due to recurrent myositis flares, intravenous immunoglobulin (IVIg) therapy was initiated in 2019. In January 2020, she underwent a thymectomy, and a histological analysis confirmed persistent thymus and a small paraganglioma. In the months following surgery, she developed fatigable ptosis, facial muscle weakness, and limb muscle fatigue. During this period, CK levels remained normal. Increasing the pyridostigmine dose relieved her symptoms. Her myasthenia symptoms have remained stable since. Due to side effects, cyclosporine was replaced with azathioprine in 2021 but was later switched to mycophenolate mofetil in 2022 due to a lack of efficacy. In 2024, rituximab was initiated due to active myositis and HRCT-confirmed pulmonary fibrosis. Currently, she is in remission on 3 × 60 mg pyridostigmine, 8 mg methylprednisolone, 2 × 1000 mg mycophenolate, 60 g IVIg per month, and rituximab.

#### 3.1.2. Patient No. 2

A 70-year-old female had a 4-year history of proximal lower extremity weakness, along with fluctuating bilateral ptosis and mild dysphagia for approximately 3 years. Serological testing revealed positive AChR antibodies (4.08 nmol/L). Her symptoms were partially relieved with pyridostigmine. Laboratory findings showed persistently elevated CK levels (~600 U/L). An EMG demonstrated myopathic changes. Alpha-glucosidase activity was normal, and the lactate stress test was negative. A limb MRI revealed fibro-fatty replacement in the gluteal and adductor muscles. A vastus lateralis muscle biopsy confirmed muscular dystrophy. Genetic screening for limb-girdle muscular dystrophy (LGMD) hotspot mutations (SGCG, FKRP, and CAPN3 genes) was negative. Gene panel testing for LGMD is in progress.

#### 3.1.3. Patient No. 4

A 59-year-old male with diabetes mellitus, diabetic polyneuropathy, RNS-confirmed AChR antibody negativity, and late-onset generalized myasthenia gravis (LOMG) was also diagnosed with anti-GAD65 antibody-positive stiff-person syndrome, affecting the distal upper extremities and anterior tibialis muscles. His CK levels were elevated (393–544 U/L). A chest CT scan revealed thymic hyperplasia. Serological testing for anti-muscle antibodies was negative. An EMG demonstrated a myogenic lesion, and a vastus lateralis muscle biopsy showed signs of myopathy (both performed under a medium-dose steroid treatment). A limb MRI, performed due to progressive weakness of the proximal upper limb muscles, revealed myositis.

#### 3.1.4. Patient No. 5

A 60-year-old male with AChR antibody-positive (119.3 nmol/L) generalized myasthenia (LOMG) presented with significantly elevated CK levels (649–1008 U/L). Increasing steroid doses led to a significant CK reduction. No additional autoimmune diseases or anti-muscle antibodies were identified. An EMG performed in 2011 showed signs of chronic myositis. However, a limb MRI (2018) and a muscle biopsy (deltoid muscle, 2012) were negative, though both were conducted under steroid therapy. In 2023, reducing methylprednisolone to 4 mg/day resulted in muscle pain and weakness. The EMG at that time revealed myositis in the anterior tibial muscle.

#### 3.1.5. Patient No. 9

A 64-year-old male was diagnosed with AChR-positive (>8 nmol/L) bulbar myasthenia, presenting with transient swallowing and articulation difficulties. An RNS and an SF-EMG confirmed myasthenia gravis. A chest CT scan showed no thymic pathology. His CK levels were moderately elevated (253–433 U/L). An EMG, a limb MRI, and MSA/MAA antibody testing were negative. CK levels remained elevated despite a temporary steroid course. Based on the IIM online calculator, inflammatory myopathy was considered unlikely; therefore, a muscle biopsy was not performed. No other autoimmune diseases were detected.

### 3.2. MSA/MAA Antibodies

In our study, 19 patients were positive for MSA or MAA (18.8%, Table 3). Myositis-specific antibodies were found in seven patients: anti-PL-7 was positive in two patients, anti-Mi-2α in one patient, anti-Mi-2β in four patients, and anti-Jo-1 in one patient. Thirteen patients harbored myositis-associated antibodies: five patients had anti-Ro-52, six patients had anti-PM-Scl-100, one patient had anti-PM-Scl-75, and four of them had anti-Ku antibodies. Multiple antibodies were detected in four patients. Serum CK levels were elevated only in the case of patient No. 1, while all the other patients had a normal result. An EMG was performed in 15 cases, confirming myositis in patient No. 1 and showing myopathy in four cases (patient No. 13, 16, 23, and 26). An MRI examination performed in five patients concluded with normal results in four cases and confirmed myositis in patient No. 1. A muscle biopsy was performed in four patients. In addition to myositis confirmed in patient No. 1, a muscle biopsy suggested myopathy in patient No. 13 during the primary diagnostic workup in 1980, and it yielded normal results in patients No. 17 and 25. Seven of the 19 MSA/MAA antibody-positive patients had an associated autoimmune disease (Sjögren’s syndrome, Raynaud’s syndrome, rheumatoid arthritis, autoimmune thyroiditis, and non-differentiated collagenosis). Except for patient No. 1, the online calculator did not suggest IIM in the other patients.

A comparison of the MGC scores of patients with or without MSA/MAA antibodies did not show a significant difference between these groups (median [IQR]: 4.0 [2.0–6.5] vs. 4.0 [1.0–7.0], respectively, *p* = 0.8296).

### 3.3. Serum Vitamin D Levels

Serum vitamin D levels were measured in all patients. Thirty-three patients (32.7%) had normal vitamin D levels, and 68 patients (67.3%) had low vitamin D levels (<75 μmol/L). Vitamin D levels were low in five out of 10 patients with elevated CK levels (Table 2), five out of seven patients with MSA antibodies, and seven out of 19 MAA-positive patients. A comparison of the MGC scores of patients with low or normal vitamin D levels did not show a significant difference between groups (median [IQR]: 4.0 [2.0–6.5] vs. 3.5 [1.5–5.8], respectively, *p* = 0.514).

## 4. Discussion

Although several case reports and small case series have reported an association between MG and IIM [16], data on the prevalence of elevated CK levels and inflammatory myopathies among MG patients treated in a tertiary center remain scarce. The prevalence of IIM among MG patients was 0.9% in a Japanese study of 924 patients [23], 2.3% in a study from Portugal [4], and 2.9% in an Italian study of 441 patients [3]. Though 9.9% of MG patients in our cohort had elevated CK levels upon entering the study, myositis could be confirmed only in one case, and probable myositis was suggested in two other cases (a total of 2.9% in our cohort). All three patients were positive for anti-AChR antibodies. In one patient with consistently elevated CK levels and clinical suspicion of a coexisting inherited muscle disease (LGMD), the muscle biopsy confirmed concomitant muscular dystrophy. Her detailed genetic examination is in progress.

Based on the data from a systematic review of MG-IIM overlap in the literature, more than 60% had an underlying thymoma [16]. In our study, two patients had thymic hyperplasia, and no thymic pathology was detected in the third case. MG was commenced 4 years earlier than the IIM in one patient (LOMG), and, similar to the literature [3], the two diseases appeared simultaneously in two other patients (one EOMG and one LOMG). According to several reports, the prevalence of MSA/MAA in MG patients with IIM is low [1,3,4,6,16,23].

Our patient with definitive PM harbored anti-Jo-1, PM/Scl-75, and Ro52 antibodies. In her case, symptoms of the anti-synthetase syndrome are dominant, putting her under strict immunological control. Up to now, only two cases with anti-Jo-1 antibody-positive MG-IIM overlap have been published. Diaco et al. reported a 47-year-old woman with anti-AChR positive MG and thymic hyperplasia who developed anti-Jo-1 antibody-positive anti-synthetase syndrome with IIM. She reacted well to prednisone and cyclosporine [24]. Huang et al. reported a 74-year-old woman with anti-AChR antibody-positive MG, IIM, and squamous cell carcinoma of the thymus. She harbored anti-Jo-1, anti-Ro52, anti-titin, and anti-ryanodine receptor antibodies. She reacted well to plasma exchange and tacrolimus [23]. Our other two patients with possible myositis and all other patients with elevated CK levels did not harbor MSA/MAA, but one patient with possible myositis had anti-GAD65 antibodies with typical signs of stiff-person syndrome. None of our MG-IIM patients harbored malignancies.

Recently, the clinical utility of MSA detection and, to a lesser extent, MAA detection has been highly acknowledged by IIM experts. In 2017, the European League Against Rheumatism (EULAR) and the American College of Rheumatology (ACR) presented new EULAR/ACR classification criteria for adult and juvenile IIM patients [22]. They recommended screening for anti-Jo-1 antiboties. An informational and instructional webpage and online calculator were also developed in which an anti-Jo-1 antibody was included. In 2018 and 2020, European Neuromuscular Centre (ENMC) experts revised their mainly clinico-pathological-oriented, expert-based classification of IIM, and clinico-seropathological-oriented updates were published for immune-mediated necrotizing myopathy (IMNM) and DM [25,26]. According to this, in the case of a characteristic clinical presentation, the presence of anti-SRP and anti-HMGCR autoantibodies for IMNM and anti-Mi2, anti-NXP2, anti-MDA5, anti-SAE, or anti-TIF1γ for DM renders a muscle biopsy optional.

Although the presence of MSA/MAA was investigated in patients with MG/IIM overlap several times, the prevalence of these antibodies among myasthenic patients is unknown. Surprisingly, we detected MSA/MAA in 18.8% of MG patients. Apart from the patient with definitive PM, all MSA/MAA-positive patients had normal CK values, and myositis could not be confirmed in these cases. A total of 37% of them (mainly those with high antibody levels) had an associated systemic autoimmune disease (e.g., Sjögren’s syndrome, Raynaud’s syndrome, rheumatoid arthritis, autoimmune thyroiditis, and non-differentiated collagenosis). In contrast, no systemic autoimmune disease was uncovered in the case of a patient with high-titer anti-Pl-7 antibodies. Two years after entering this study, she was diagnosed with diffuse large B-cell lymphoma (DLBCL). Another patient with a high anti-Ro52 antibody titer had no known systemic autoimmune disease.

A total of 63% of our MSA/MAA-positive cases had low-titer antibodies (anti-Ku, anti-Mi2β, anti-PM-Scl-100, or anti-Pl-7). One of the patients with anti-Ku positivity had Raynaud’s syndrome, while another with anti-PM-Scl-100 antibodies had thyroiditis. Systemic autoimmune diseases (including IIM) could not be validated in the other patients. Multiple antibodies were not detected. The significance of low-titer MSA/MAA antibodies in our patients is questionable. According to a study of 727 patients with different neurological diseases, MAA and MSA may also occur in approximately 6.7% to 30.0% of cases [27]. In their opinion, the relatively high frequency of false-positive antibodies should be considered in clinical practice. Beyond that, several studies have demonstrated that line/dot blot assays suffer from limited specificity [28], since 9–16% of healthy controls have been found to be positive for MSA/MAA with different line blot assays [29,30,31]. In these cases, false-positive results were generally low-titer, and false-positive samples more often showed multiple autoantibody positivity. Validation, standardization, and harmonization of the MSA/MAA tests have been suggested to improve the clinical utility of these assays [28].

The role of vitamin D in immunomodulation has been extensively studied. The loss of tolerance to self-antigens occurs in many autoimmune diseases and appears to be associated with lower vitamin D levels [9,32]. It is also known that vitamin D receptors are found in muscle cells, playing a role in muscle regeneration, with their deficiency resulting in muscle weakness and myalgia [9]. Therefore, vitamin D is thought to have a dual effect on MG by regulating the autoimmune response and maintaining muscle function. One study of 151 MG patients showed an association between the risk and characteristics of myasthenia and vitamin D receptor gene polymorphism [33]. Several publications have reported lower serum vitamin D levels in patients with myasthenia [9,11,12,13,14]. A meta-analysis of five studies, including a total population of 219 MG patients and 231 normal control cases, confirmed this observation, suggesting routine checks and possible correction of vitamin D levels in MG patients [34]. By contrast, a two-sample Mendelian randomization study found no evidence that circulating vitamin D impacts the risk of developing MG [35]. From the point of view of treatment, it is important to mention that cholecalciferol supplementation (800 IU/day) in MG patients with low vitamin D levels improves muscle weakness [9]. In this study, we determined the cholecalciferol levels in a large population of MG patients in East-Central Europe. Consistent with the literature, we found low vitamin D levels in 67.3% of our MG patients. MGC scores of patients with normal and low vitamin D levels did not differ significantly. A follow-up study after normalizing cholecalciferol levels should be performed in the future to judge the effect of vitamin D on the quality of life of patients with MG.

In conclusion, the coexistence of myasthenia gravis and idiopathic myositis is a rare but clinically significant phenomenon which may affect roughly 1–3% of MG patients [3,4,23]. It is recommended to assess CK levels in MG patients with muscle pain, non-fatigable muscle weakness, or in case of a phenotype suspicious for hereditary myopathy or muscular dystrophy. When high (>2× ULN) CK levels are confirmed or there is a continuous need for steroid therapy, further examinations should be performed for overlapping muscular diseases, including myositis. In the case of a slight increase in CK levels (<2× ULN) discovered by chance, identifying predisposing factors (e.g., thyroid gland disease, use of statins) and follow-up to monitor CK levels are suggested (e.g., once or twice monthly). Testing of MSA/MAA antibodies does not appear to be a suitable primary screening method for IIM, as such antibodies are associated with a relatively high rate of false, low-titer, positive results. Our findings also emphasize the importance of regular vitamin D level assessments and supplementation in managing MG. Finally, it is worth keeping in mind that the MG population is not a homogeneous cohort, as these patients may (even if not often) have other neuromuscular diseases (e.g., muscular dystrophies).

### Limitations

This study has several limitations. First, the sample size was relatively small, limiting the generalizability of the findings. Second, the study was conducted on a Caucasian population in East-Central Europe, an approach that may restrict the applicability of our results to more diverse populations. However, it is important to note that genetic variability, rather than ethnicity alone, may influence the clinical phenotype of myasthenia gravis, and genetic analysis was not performed in this study. Third, our study design lies between a cross-sectional study and a case series, a design that inherently carries the risk of selection and observational bias. The retrospective component of the study may have introduced data collection biases. Some clinical parameters, as well as comprehensive antibody testing for MG-specific antibodies, were not available for all patients. The non-randomized, sequential enrollment of patients from a single tertiary neuromuscular center may have also introduced sampling bias, potentially limiting the external validity of our findings. Fourth, vitamin D levels were measured at a single time point upon admission to the study. This cross-sectional approach provides only a snapshot of vitamin D status and does not account for potential fluctuations over time, seasonal variations, or potential impacts on disease progression. In addition, we did not systematically assess vitamin D supplementation among patients. Longitudinal studies are needed to determine the impact of vitamin D levels and supplementation on disease severity.

## Figures and Tables

**Table 1:** Baseline characteristics of the participants.

Characteristic	Value (*N* = 101)
**Age in years (median, IQR)**	57 (45–70)
**Age in years at MG Onset (median, IQR)**	43 (30–57)
**Sex**	
Male	27 (26.7%)
Female	74 (73.2%)
**MG subtype**	
Generalized	68 (67.33%)
Bulbar	18 (17.82%)
Ocular	15 (14.85%)
**AChR antibody positive**	67 (66.3%)
**MuSK antibody positive**	4 (4.0%)
**Seronegative MG**	14 (13.9%)
**Patients without antibody testing**	16 (15.8%)
**Thymoma**	6 (5.9%)
**Thymic hyperplasia**	39 (38.6%)
**Disease duration in years (median, IQR)**	7 (2–17)

MG: myasthenia gravis, AChR: acetylcholine receptor, MUSK: muscle-specific kinase, IQR: interquartile range.

**Table 2:** Characteristics of patients with elevated serum CK levels.

No.	Gender	Age at Onset of MG/IIM	Type of MG	Antibody	RNS	SF-EMG	Thymus	MGFA Score	MGC	CK (U/L)	MSA/ MAA/ Other Ab	Vitamin D (nmol/L)	EMG	MRI	Biopsy
1	F	28/28	G	AChR	Norm.	nd	Hyperplasia	IIA	7	916..512	Jo, Ro52, PM/Scl-75	138	Myositis	Myositis	Myositis (vastus lat.)
2	F	65/	G	AChR	Norm.	nd	Normal	IIA	9	591..604	Neg.	70.2	Myopathy	Fibro-fatty replacement	Dystrophy (vastus lat.)
3	F	24/	G	nd	Decr.	nd	Hyperplasia	IIA	7	266..norm.	Neg.	68.8	Myopathy	nd	nd
4	M	51/56	G	Neg.	Decr.	nd	Hyperplasia	IIA	8	393..544	GAD	104	Myopathy	Myositis	Myopathy (vastus lat.)
5	M	50/50	G	AChR	Decr.	nd	Normal	0	0	649..964	Neg.	50.2	Myositis	Normal	Normal (deltoid)
6	F	66/	G	Neg.	Decr.	Positive	Normal	0	0	210..norm.	Neg.	82	Normal	nd	nd
7	F	74/	G	AChR	Decr.	Positive	Normal	I	3	359..norm.	Neg.	104	Normal	nd	nd
8	F	21/	G	AChR	Decr.	Positive	Hyperplasia	IIA	3	268..306	Neg.	69	Normal	nd	nd
9	M	62/	B	AChR	Decr.	Positive	Normal	IIB	2	253..433	Neg.	100	Normal	Normal	nd
10	M	39/	G	AChR	nd	nd	Normal	IIA	4	263..257	Neg.	15.7	Normal	nd	nd

Ab: antibody, M: male, F: female, G: generalized, B: bulbar, Decr.: decrement, Neg.: negative, nd: not done, AChR: acetylcholine receptor.

**Table 3:** Characteristics of patients with positive serum MSA/MAA levels.

No.	Gender	Age at onset of MG	Type of MG	MG Ab	RNS	SF- EMG	Thymus	MGFA Score	MGC	CK (U/L)	MSA/ MAA/ Other Ab	Vitamin D (nmol/L)	EMG	MRI	Biopsy	Associated ID
1	F	28	G	AChR	Norm.	nd	Hyperplasia	IIA	7	916..512	Jo (+++), PM-Scl 75 (+), Ro52 (++)	138	Myositis	Myositis	Myositis (vastus lat.)	Anti-synthetase syndrome
11	F	37	G	AChR	Norm.	nd	Normal	IIA	6	Norm.	Ro-52 (+++), PM-Scl-100 (++)	94.5	Normal	nd	nd	Sjögren sy, Raynaud sy
12	F	52	G	nd	Decr.	Positive	Hyperplasia	IIA	12	Norm.	PM-Scl-100 (+), Ku (++)	63.8	Normal	Normal	nd	Polyarthritis
13	F	31	G	nd	Decr.	Positive	Hyperplasia	IIA	5	Norm.	PL-7 (+++)	72	Myopathy	nd	Dystrophy (vastus lat.)	No
14	F	31	O	AChR	Decr.	Positive	Normal	I	3	Norm.	Mi-2β (+)	77.8	Normal	Normal	nd	No
15	F	37	G	neg	Decr.	nd	Normal	0	0	Norm.	Ku (+)	67.5	Normal	Normal	nd	Raynaud
16	F	54	G	AChR	nd	Positive	Thymoma	IIA	2	Norm.	Ku (+)	91.2	Mild myopathy	Normal	nd	No
17	F	15	G	AChR	nd	nd	Hyperplasia	IIA	8	Norm.	PM-Scl-100 (+)	33.2	Normal	nd	Normal (deltoid)	No
18	F	51	B	AChR	Decr.	nd	Normal	IIA	6	Norm.	Ro-52 (+)	106	Normal	nd	nd	No
19	F	40	G	nd	Norm.	nd	Hyperplasia	IIA	8	Norm.	Mi-2β (+)	11.8	nd	nd	nd	No
20	M	78	O	AChR	Norm.	nd	Normal	IIA	2	Norm.	PM-Scl-100 (+)	25	nd	nd	nd	Thyreoiditis
21	F	20	G	nd	Decr.	nd	Hyperplasia	IIA	1	Norm.	Ro-52 (+++)	59.5	Normal	nd	nd	Sjögren, Raynaud, RA
22	F	21	G	AChR	nd	Positive	Hyperplasia	0	0	Norm.	Mi-2β (+)	66.8	Normal	nd	nd	No
23	F	30	G	AChR	Decr.	nd	Normal	IIA	2	Norm.	Ku (+)	26.2	Mild myopathy	nd	nd	No
24	F	36	G	AChR	nd	nd	Hyperplasia	IIA	4	Norm.	Pl-7 (+)	51.5	Normal	nd	nd	No
25	F	25	B	MUSK	Decr.	Negative	Normal	IIA	5	Norm.	PM-Scl-100 (+)	42.2	Normal	Normal	Normal (deltoid)	No
26	F	27	G	AChR	nd	nd	Hyperplasia	IIA	9	Norm.	PM-Scl-100 (++)	115.5	Myopathy (steroid?)	nd	nd	NDC, Raynaud sy., Polyarthritis
27	M	19	O	AChR	Norm.	Positive	Normal	0	0	Norm.	Mi-2β (+)	79.2	nd	nd	nd	No
28	M	82	O	AChR	nd	Positive	Normal	I	7	Norm.	Ro-52 (+++)	81	nd	nd	nd	No

ID: immune disease, Ab: antibody, M: male, F: female, G: generalized, B: bulbar, O: ocular, Decr.: decrement, nd: not done, Norm.: normal.

## Data Availability

The data supporting this study’s findings are available from the corresponding author upon reasonable request.

## Abbreviations

The following abbreviations are used in this manuscript:

AChR	acetylcholine receptor
ACR	American College of Rheumatology
CK	creatine kinase
CT	computed tomography
DLBCL	diffuse large B-cell lymphoma
DM	dermatomyositis
EOMG	early-onset myasthenia gravis
EMG	electromyography
ENMC	European Neuromuscular Centre
EULAR	European League Against Rheumatism
GAD65	glutamic acid decarboxylase 65
HRCT	high-resolution computed tomography
IBM	inclusion body myositis
ICI	immune-checkpoint inhibitor
IQR	interquartile range
IIM	idiopathic inflammatory myopathy
IMNM	immune-mediated necrotizing myopathy
IU	international units
IvIg	intravenous immunoglobulin
LGMD	limb-girdle muscular dystrophy
LOMG	late-onset myasthenia gravis
LRP4	lipoprotein receptor-related protein 4
MAA	myositis-associated antibodies
MG	myasthenia gravis
MGC	myasthenia gravis composite
MGFA	myasthenia gravis foundation of America
MRI	magnetic resonance imaging
MS	multiple sclerosis
MSA	myositis-specific antibodies
MUSK	muscle-specific kinase
PM	polymyositis
RA	rheumatoid arthritis
RNS	repetitive nerve stimulation
Ro-52	anti-Ro52 antibody (specific autoantibody)
SF-EMG	single-fiber electromyography
SLE	systemic lupus erythematosus
STIR	short tau inversion recovery
ULN	upper limit of normal
